# A reproducible mechano-thermal injury method for reconstructed human epidermis to study wound re-epithelialization

**DOI:** 10.1016/j.mex.2026.103976

**Published:** 2026-05-26

**Authors:** Thaís C. Pereira, Victória S. Padilha, José M. de Brito Neto, Raquel F. Chaves, Vanja Dakic, Ronaldo José F․ C․ do Amaral

**Affiliations:** a L’Oréal Research & Innovation, Rio de Janeiro, Brazil; b Institute of Biomedical Sciences, Federal University of Rio de Janeiro (UFRJ), Rio de Janeiro, Brazil; c Postgraduate Program in Medicine (Pathological Anatomy), School of Medicine, Federal University of Rio de Janeiro, Rio de Janeiro, Brazil; d EPISKIN, Rio de Janeiro, Brazil; e University of Grande Rio (Unigranrio), Rio de Janeiro, RJ, Brazil

**Keywords:** RHE, SkinEthic®, Re-epithelialization, *In vitro* assay

## Abstract

Reconstructed human epidermis (RHE) models are widely used as an ethical alternative to animal testing and are validated for cutaneous toxicity assessment. However, their application in wound healing research remains limited due to the lack of standardized methods to induce reproducible epidermal injuries. Generating localized lesions while preserving the polycarbonate membrane required for keratinocyte migration remains a technical challenge. Here we describe a method to generate controlled lesions in reconstructed human epidermis suitable for re-epithelialization studies. Several injury strategies were evaluated, including mechanical approaches using pipette tips, punches, microneedles and glass capillaries. These approaches frequently produced inconsistent lesions or disrupted the membrane. A mechano-thermal strategy proved to be the most reliable approach, generating well-defined and reproducible lesions while preserving membrane integrity. Histological analysis confirmed characteristic features of thermal injury. Longitudinal macroscopic and histological analyses, including semi-quantitative scoring, demonstrated progressive epidermal reorganization following injury. This method establishes a reproducible epidermal burn model suitable for studying epidermal regeneration and wound healing.

• Comparative evaluation of mechanical and mechano-thermal strategies to induce lesions in reconstructed human epidermis.
• Identification of a reproducible injury method that preserves the polycarbonate membrane required for keratinocyte migration.
• Development of a standardized epidermal burn model for re-epithelialization studies.

## Graphical abstract

**Figure:**
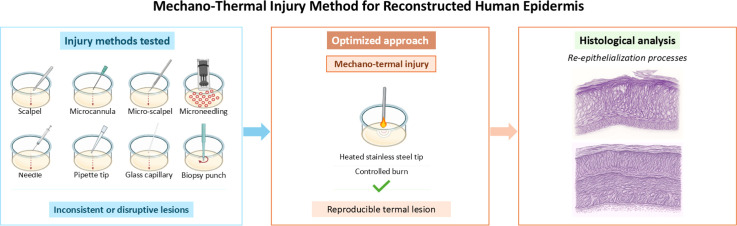

**Specifications table**

**Table:** 

**Subject area**	Medicine and Dentistry .
**More specific subject area**	Wound Healing.
**Name of your method**	Mechano-thermal injury method for reconstructed human epidermis.
**Name and reference of original method**	A similar mechano-thermal approach has been reported in the literature [1]. Here, the method was independently developed through systematic testing of multiple injury strategies in reconstructed human epidermis. This study not only describes a suitable approach but also documents unsuccessful attempts and methodological limitations. Sharing these observations is intended to support reproducibility and reduce trial-and-error in future applications of this model.
**Resource availability**	The method requires reconstructed human epidermis (RHE) models and a stainless-steel tip for controlled mechano-thermal injury induction. The tip is heated using a heating plate and its temperature is monitored with an infrared thermometer to ensure consistent application (80 °C). The heated tip is applied to the epidermal surface for 3 s to induce a reproducible lesion. Standard histological processing equipment is required for tissue analysis, and a stereomicroscope is recommended for lesion visualization and monitoring.

## Background

The skin, the largest organ of the human body, acts as the first line of defense and plays a key role in maintaining fluid balance and body temperature, thereby ensuring homeostasis [2,3]. It is composed of the epidermis and dermis, functionally associated with the subcutaneous tissue. The epidermis, the outermost layer, consists primarily of keratinocytes, which undergo a continuous process of proliferation, differentiation, and migration known as keratinization [4]. Due to its direct exposure to the external environment, the skin is highly susceptible to injury [5].

Animal models have traditionally been used to study wound healing; however, their translational limitations and ethical concerns have driven the development of alternative approaches [6]. Regulatory advances, including the European ban on animal testing for cosmetics (EU Regulation 1223/2009) and similar restrictions in Brazil, have reinforced the need for reliable *in vitro* models. In this context, reconstructed human epidermis (RHE) models have emerged as robust and reproducible alternatives [7].

The SkinEthic® RHE model, generated from human keratinocytes cultured at the air–liquid interface, is widely used in validated assays for skin irritation and corrosion (OECD TG 431 and 439) and other applications [8,9]. Despite this, its potential for wound healing studies remains underexplored. RHE models could serve as valuable platforms for screening compounds that modulate re-epithelialization, a critical process in tissue repair.

Most wound healing studies rely on full-thickness skin models, while comparatively few focus exclusively on the epidermis. However, RHE models offer important advantages, including a targeted analysis of keratinocyte behavior, improved experimental control, and enhanced reproducibility. Additionally, their simplified structure and cost-effectiveness make them suitable for large-scale applications.

Importantly, existing studies often assume that lesion induction in RHE is straightforward, providing limited methodological detail. In practice, achieving controlled and reproducible epidermal injury presents significant technical challenges, which may compromise experimental consistency and limit broader application of the model.

Therefore, this study aims to establish a simple, low-cost, and reproducible mechano-thermal injury method in SkinEthic® RHE, providing a practical framework to support re-epithelialization studies and reduce trial-and-error in future applications.

## Method details

### Overview

**Fig. 1:**
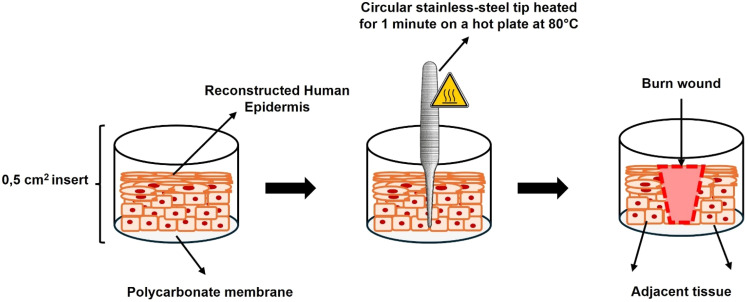
**Schematic overview of the mechano-thermal injury method in reconstructed human epidermis (RHE).** A stainless-steel tip is heated for 1 min (80 °C) and applied for 3 s to the epidermal surface to generate a localized burn lesion while preserving the polycarbonate membrane.

**Fig. 2:**
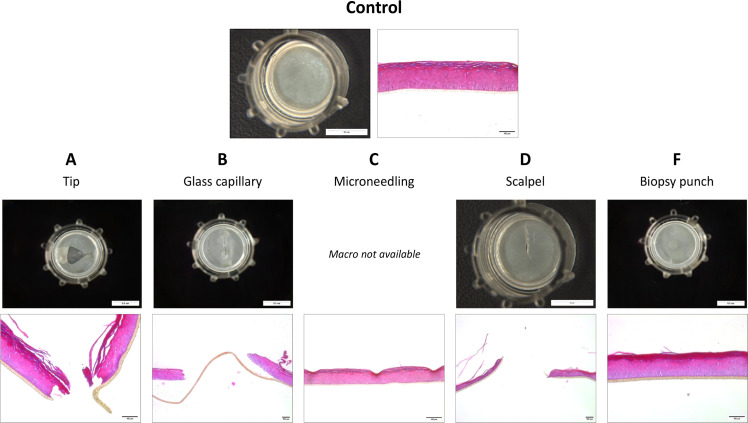
**Comparative evaluation of mechanical injury methods in reconstructed human epidermis (RHE).** Representative macroscopic (top row) and histological images (bottom row, H&E staining) of lesions induced using different mechanical approaches: (A) pipette tip, (B) glass capillary, (C) microneedling, (D) scalpel, and (F) biopsy punch. Control (non-injured) tissue is shown above for reference. Macroscopic images were not available for microneedling due to the need to remove the tissue from the insert for device application. Macroscopic images were acquired using different stereomicroscopes; however, identical scale bars are provided for comparison. Scale bars in histological images may vary. Images are representative of independent experiments (*n* = 3).

This protocol describes a mechano-thermal approach to induce controlled and reproducible lesions in reconstructed human epidermis (RHE). The method is based on the application of a heated stainless-steel tip to the epidermal surface, allowing the generation of localized thermal injury while preserving the integrity of the underlying polycarbonate membrane. This approach was established after evaluating multiple mechanical and mechano-thermal strategies. (Figs. 1 and 2).

### Materials

• Reconstructed human epidermis (RHE) model (*e.g.*, SkinEthic® RHE).
• Stainless-steel tip (flat surface).
• Heating plate.
• Infrared thermometer.
• Stereomicroscope.
• Standard cell culture equipment.
• Histology reagents and processing materials.

### Method step-by-step

#### Step 1 – preparation of RHE

RHE tissues are removed from their packaging and transferred to 6-well plates containing 300 μl of growth medium (SGM, provided by the supplier) and equilibrated for 2 h at 37 °C in a humidified incubator with 5% CO_2_ prior to injury induction.

#### Step 2 – heating of the stainless-steel tip

The stainless-steel tip is placed on a heating plate and heated until reaching 80 °C. The temperature is monitored using an infrared thermometer immediately prior to application to ensure consistency.

#### Step 3 – injury induction

The heated tip is gently applied to the center of the epidermal surface for 3 s. Care is taken to apply minimal pressure to avoid disruption of the polycarbonate membrane. The tip is removed vertically after contact.

#### Step 4 – post-injury handling

Following injury induction, tissues are maintained under standard culture conditions. The culture medium (SGM) is refreshed every 24 h using 1 ml per well. If a 48 h interval is used, the medium volume is increased to 2 ml to ensure adequate nutrient supply and tissue viability. Samples can be collected at defined time points depending on the experimental design.

#### Step 5 – tissue analysis

Macroscopic images of the lesions were acquired at different timepoints using a stereomicroscope. Lesion areas were quantified using ImageJ software (National Institutes of Health, USA). Briefly, the lesion boundaries were manually delineated using the freehand selection tool, and the corresponding lesion area was calculated after image calibration using the scale bar, with results expressed in mm².

For histological evaluation, H&E-stained sections were analyzed for morphological alterations associated with injury and tissue regeneration. Histological alterations were semi-quantitatively scored by two blinded observers using a four-point scale (0–3), where 0 = absent, 1 = mild, 2 = moderate, and 3 = severe. Parameters evaluated included epidermal disorganization, cytoplasmic vacuolization, epidermal detachment, and re-epithelialization.

### Critical points

• The temperature of the stainless-steel tip must be maintained at 80 °C to ensure reproducibility.
• Excessive pressure during application may disrupt the polycarbonate membrane and compromise the model.
• Contact time shorter than 3 s may result in inconsistent or insufficient injury.
• Temperature fluctuations between applications may affect lesion size and depth.

### Troubleshooting and methodological considerations

Several mechanical approaches were systematically evaluated during method development, revealing important technical limitations that are not always explicitly reported in literature. These observations, although not all shown in figures, were critical for refining the final protocol.

Sharp instruments, including needles, scalpels, and micro-scalpels, consistently resulted in uncontrolled tissue disruption and frequent rupture of the underlying polycarbonate membrane. These approaches lacked precision and reproducibility, making them unsuitable for generating standardized epidermal lesions.

The use of microcannulas led predominantly to detachment of the epidermis from the membrane without inducing a defined lesion, compromising the structural integrity of the model and preventing meaningful analysis of re-epithelialization.

Pipette tips produced highly variable outcomes, ranging from excessively large and irregular lesions to complete membrane rupture or epidermal detachment, depending on the applied force and tip geometry. Similarly, glass capillaries generated extensive and poorly controlled lesions, limiting their applicability for reproducible injury induction.

Microneedling approaches were also investigated using different needle depths. However, due to the size constraints of the device, it was necessary to remove the tissue from the insert prior to application. This disrupted the experimental setup and prevented longitudinal macroscopic monitoring of the same sample, thereby limiting the assessment of re-epithelialization over time.

Biopsy punch application revealed a strong dependence on the applied pressure. Minimal force resulted only in superficial markings without effective lesion formation, whereas increased pressure frequently caused damage to the underlying polycarbonate membrane. This narrow operational window made the method difficult to standardize and poorly reproducible.

Altogether, these findings demonstrate that purely mechanical approaches are highly sensitive to operator-dependent variables and often fail to generate controlled and reproducible lesions in reconstructed human epidermis models. Reporting these limitations is essential to inform the scientific community and to reduce unnecessary trial-and-error in future applications.

These limitations directly motivated the development of a controlled mechano-thermal approach, designed to overcome the inconsistencies observed with purely mechanical methods.

### Expected outcomes

When properly performed, this protocol yields a localized and reproducible epidermal lesion characterized by well-defined boundaries. Histological analysis typically reveals features consistent with thermal injury, including epidermal detachment, pyknotic nuclei, and cellular swelling. Importantly, the integrity of the polycarbonate membrane is preserved, enabling subsequent analysis of re-epithelialization processes.

### Potential applications and future perspectives

Although reconstructed human epidermis (RHE) models are widely validated for skin irritation and corrosion testing according to OECD Test Guidelines 431 and 439 [[10], [11], [12], [13]], their application in wound healing and re-epithelialization studies remains comparatively underexplored. Most currently available wound healing studies employ full-thickness or organotypic skin equivalents rather than epidermis-only models [14,15]. However, these simplified systems may provide important advantages for investigating epidermal-specific regenerative events, including keratinocyte migration, proliferation, and differentiation [16,17].

The present work demonstrates that establishing reproducible lesions in RHE models is not trivial and may involve important technical limitations depending on the injury approach employed. Many previous studies focus primarily on downstream biological analyses without extensively discussing the practical challenges associated with lesion induction, especially in reconstructed epidermis models where preservation of the polycarbonate support membrane is essential for re-epithelialization analyses [1]. In addition, some wound healing studies employing full-thickness or organotypic skin models rely on specialized equipment, such as laser-based injury systems [18,19], which may limit accessibility and implementation across different laboratories. Therefore, the present study aimed to establish a simple, low-cost, and broadly applicable mechano-thermal injury method using easily accessible laboratory materials. Nevertheless, different injury methods may be appropriate depending on the specific experimental objective, model configuration, and downstream analyses intended. By systematically documenting the different approaches evaluated throughout method development, this study aims to provide practical guidance that may help other researchers avoid time-consuming trial-and-error procedures.

The proposed mechano-thermal injury method may support future applications involving the screening of soluble compounds, cosmetic ingredients, biomaterials, or regenerative strategies capable of modulating epidermal repair [16,20,21]. In addition, the model may be useful for studies investigating re-epithelialization kinetics, epidermal regeneration, and histological responses following injury in a controlled *in vitro* environment.

Ongoing studies aim to expand the biological characterization and functional applications of this model. In addition, the applicability and reproducibility of the proposed method across different reconstructed epidermis platforms and culture conditions may also be explored in future investigations.

## Method validation

**Fig. 3:**
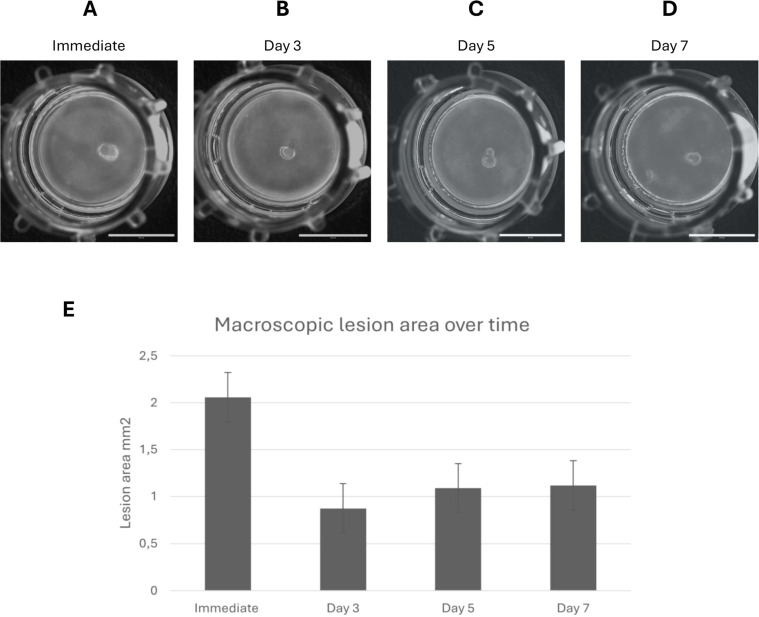
**Macroscopic evaluation of mechano-thermal injury progression in reconstructed human epidermis (RHE).** (A–D) Representative macroscopic images of reconstructed human epidermis immediately after injury induction and during the re-epithelialization process at days 3, 5, and 7 post-injury. Lesions remained localized and progressively reduced in size over time while preserving membrane integrity. Scale bars = 0.5 cm. (E) Quantification of macroscopic lesion area over time, expressed in mm². Data are presented as mean ± SD (*n* = 3).

The mechano-thermal approach enabled the reproducible induction of localized epidermal lesions while preserving the integrity of the polycarbonate support membrane. Immediately after injury, lesions appeared macroscopically well-defined and homogeneous across samples (Fig. 3A). Longitudinal macroscopic monitoring demonstrated a progressive reduction in lesion area over time, particularly between the immediate post-injury condition and day 3, supporting the reproducibility and temporal follow-up capability of the model (Fig. 3B–E).

**Fig. 4:**
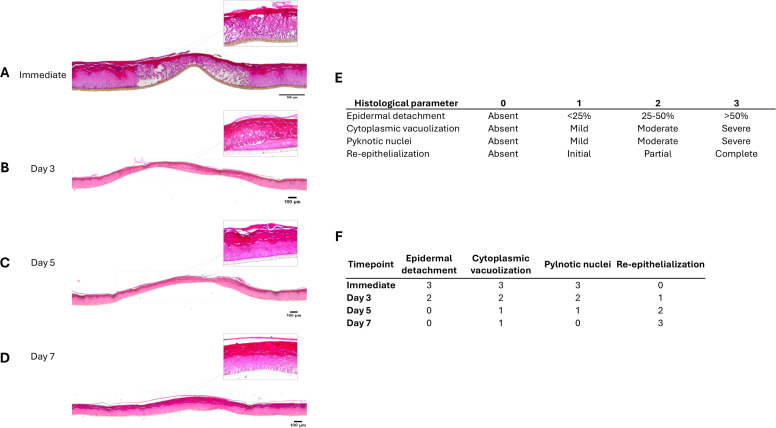
**Histological evaluation and semi-quantitative analysis of epidermal regeneration following mechano-thermal injury in reconstructed human epidermis (RHE).** (A–D) Representative hematoxylin and eosin (H&E)-stained sections immediately after injury induction and at days 3, 5, and 7 post-injury. Immediate post-injury sections showed epidermal detachment, cellular disorganization, cytoplasmic vacuolization, and pyknotic nuclei. Progressive epidermal reorganization and re-epithelialization were observed over time. Insets highlight representative histological features at each timepoint. Scale bars = 100 µm. (E) Semi-quantitative histopathological scoring system used to evaluate epidermal detachment, cytoplasmic vacuolization, pyknotic nuclei, and re-epithelialization. (F) Semi-quantitative histopathological scores obtained at each experimental timepoint.

Histological evaluation confirmed characteristic features of thermal injury immediately after lesion induction, including epidermal detachment from the membrane, pyknotic nuclei, cellular swelling, and tissue disorganization (Fig. 4A). At day 3 post-injury, the lesion area displayed immature and disorganized epidermal layers associated with marked cytoplasmic vacuolization (Fig. 4B). By day 5, partial structural recovery was observed, with improved stratification and reduced vacuolization (Fig. 4C). Finally, at day 7 post-injury, the epidermis exhibited a more organized and mature architecture, suggestive of progressive re-epithelialization (Fig. 4D).

To complement the descriptive histological analysis, a semi-quantitative histopathological scoring system was applied to evaluate tissue organization and regenerative progression over time (Fig. 4E–F). The scores obtained were consistent with the morphological observations, supporting the progressive recovery of epidermal architecture following injury.

Together, these findings demonstrate that the proposed mechano-thermal method enables reproducible lesion induction and longitudinal evaluation of epidermal regeneration in reconstructed human epidermis.

## Limitations

Although the proposed mechano-thermal approach enables reproducible and localized epidermal injury, some limitations should be considered. First, the reconstructed human epidermis (RHE) model lacks a dermal compartment and therefore does not fully recapitulate the complexity of full-thickness skin wound healing.

In addition, the method relies on precise control of temperature and application time, and minor variations in these parameters may affect lesion size and reproducibility. Similarly, manual application of the heated tip may introduce operator-dependent variability if not carefully standardized.

Another limitation of the present study is that the method was validated using a single commercially available RHE system and its corresponding supplier-provided culture medium (SGM). Therefore, additional studies may be necessary to evaluate the applicability and reproducibility of this approach in other reconstructed epidermis platforms or culture conditions.

Furthermore, the geometry and dimensions of the RHE inserts impose physical constraints that may limit the compatibility of certain injury devices or experimental approaches.

Despite these limitations, the model provides a controlled, accessible, and reproducible platform for the induction and longitudinal evaluation of epidermal injury and re-epithelialization *in vitro*.

## Ethics statements

This study did not involve human participants, animal experiments, or data collected from social media platforms. Therefore, ethical approval and informed consent were not required.

## CRediT author statement

**Conceptualization**: Thaís C. Pereira and Ronaldo José F. C. do Amaral; **Methodology**: Thaís C. Pereira, Victória S. Padilha, José M. Brito Neto and Raquel F. Chaves; **Formal analysis and investigation**: Thaís C. Pereira and Victória S. Padilha; **Writing - original draft preparation**: Thaís C. Pereira; **Writing** - **review and editing**: Thaís C. Pereira, Victória S. Padilha, José M. Brito Neto, Raquel F. Chaves, Vanja Dakic and Ronaldo José F. C. do Amaral; **Funding acquisition**: Vanja Dakic; **Resources**: Vanja Dakic and Ronaldo José F. C. do Amaral; **Supervision**: Vanja Dakic and Ronaldo José F. C. do Amaral.

## Declaration of competing interest

The authors declare the following potential competing interests: Thaís C. Pereira and Vanja Dakic are employees of L’Oréal Research & Innovation and Episkin. The remaining authors declare that the research was conducted in the absence of any commercial, financial, or personal relationships that could be construed as a potential conflict of interest.

## Data Availability

All data supporting the findings of this study are included within the article. Additional data are available from the corresponding author upon reasonable request.
